# Supplementary material for: The Korea Cancer Big Data Platform (K-CBP) for Cancer Research
Source: Int J Environ Res Public Health. 2019 Jun 28;16(13):2290. doi: 10.3390/ijerph16132290 (PMC6651426; doi:10.3390/ijerph16132290)
Supplement: Supplementary file 1 [file ijerph-16-02290-s001.zip › Supplementary Material 2.docx]

| **Cancer Type** | **URL** | **QR code** |
| --- | --- | --- |
| Pancreatic | https://doi.org/10.22694/CANCERDATA032018070101 | 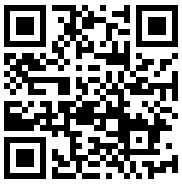 |
| Lung | https://doi.org/10.22694/CANCERDATA032018070102 | 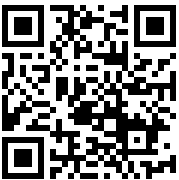 |
| Prostatic | https://doi.org/10.22694/CANCERDATA032018070103 | 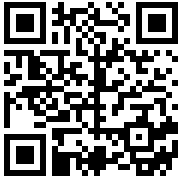 |
| Biliary tract | https://doi.org/10.22694/CANCERDATA032018070104 | 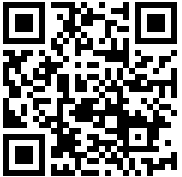 |
| Ovarian | https://doi.org/10.22694/CANCERDATA032018070105 | 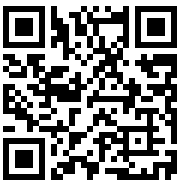 |
| Kidney | https://doi.org/10.22694/CANCERDATA032018070106 | 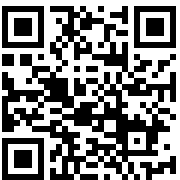 |

**Supplementary Table 2: Clinical cancer registry DOI URL and QR code**
